# *Moringa oleifera* Mitigates Memory Impairment and Neurodegeneration in Animal Model of Age-Related Dementia

**DOI:** 10.1155/2013/695936

**Published:** 2013-12-23

**Authors:** Chatchada Sutalangka, Jintanaporn Wattanathorn, Supaporn Muchimapura, Wipawee Thukham-mee

**Affiliations:** ^1^Department of Physiology (Neuroscience Program), Faculty of Medicine, Khon Kaen University, Khon Kaen 40002, Thailand; ^2^Integrative Complementary Alternative Medicine Research and Development Center, Khon Kaen University, Khon Kaen 40002, Thailand; ^3^Department of Physiology, Faculty of Medicine, Khon Kaen University, Khon Kaen 40002, Thailand

## Abstract

To date, the preventive strategy against dementia is still essential due to the rapid growth of its prevalence and the limited therapeutic efficacy. Based on the crucial role of oxidative stress in age-related dementia and the antioxidant and nootropic activities of *Moringa oleifera*, the enhancement of spatial memory and neuroprotection of *M. oleifera* leaves extract in animal model of age-related dementia was determined. The possible underlying mechanism was also investigated. Male Wistar rats, weighing 180–220 g, were orally given *M. oleifera* leaves extract at doses of 100, 200, and 400 mg/kg at a period of 7 days before and 7 days after the intracerebroventricular administration of AF64A bilaterally. Then, they were assessed memory, neuron density, MDA level, and the activities of SOD, CAT, GSH-Px, and AChE in hippocampus. The results showed that the extract improved spatial memory and neurodegeneration in CA1, CA2, CA3, and dentate gyrus of hippocampus together with the decreased MDA level and AChE activity but increased SOD and CAT activities. Therefore, our data suggest that *M. oleifera* leaves extract is the potential cognitive enhancer and neuroprotectant. The possible mechanism might occur partly via the decreased oxidative stress and the enhanced cholinergic function. However, further explorations concerning active ingredient(s) are still required.

## 1. Background

Dementia, a serious loss of global cognitive ability including the impairments of memory, attention, language, and problem solving, is continually growing worldwide accompanied with the increased elderly population. It has been estimated that there are approximately 35.6 million people with dementia worldwide [1]. Due to the rapidly growth of prevalence, high expenditure cost, and unsatisfactory outcomes of therapeutic strategy, dementia has been recognized as one of the major medical and social challenges especially in developing countries [2].

Recent findings have shown that the age-related cognitive dysfunction occurs as a result of oxidative stress elevation in the brain [3], hippocampal atrophy [4], and the disturbances of neurotransmission, especially cholinergic transmission. Therefore, the modulation of cholinergic function becomes the approach to dementia treatment. However, most drugs still induce adverse effects [5]. This disadvantage consequently motivates research effort to find out novel protective agent against dementia.

Herbal medicine has long been used to treat numerous ailments. Moreover, the “Green” movement has driven the attitude changes of the general population to prefer naturally derived substances and extracts as being inherently safer and more desirable than synthetic chemical products. Accumulative lines of evidence have demonstrated that consumption of antioxidant-rich foods and polyphenol treatment can enhance cognitive performance in elderly subjects [6–8].

*Moringa oleifera*, a plant in the family of Moringaceae, is an edible plant which has been used both as food and as medicine in many Asian countries including Thailand for centuries. The leaves of this plant have been reported to be a rich source of potassium, calcium, phosphorous, iron, vitamins A and D, essential amino acids, and antioxidants such as *β*-carotene, vitamin C, and flavonoids [9–13]. In addition, antinutrients such as alkaloids, tannins, phenolics, saponins, and steroids were also observed [14]. The leaves extract also exhibits antioxidant activity [15]. Recently, it has been shown that *M. oleifera* leaves extract at doses more than 3000 mg/kg show genotoxicity effect. In addition, LD50 of the alcoholic extract of *M. oleifera* leaves is reported to be more than 2800 mg/kg. [16]. Therefore, the leaves extract intake is safe at dose ≤ 1000 mg kg-1 BW [17]. The extract also possesses antioxidant and nootropic effects. Moreover, it has been reported to combat oxidative stress in rat model of Alzheimer's disease induced by colchicines such as vitamin C and vitamin E [18, 19]. However, the scientific evidence concerning the effect of *M. oleifera* leaves extract on cognitive dysfunction induced by hypocholinergic function, the important cause of memory impairment in dementia, is limited until now. Therefore, this study aimed to investigate the memory enhancing effect, neuroprotective effect, and possible underlying mechanism of *M. oleifera* leaves extract in animal model of dementia induced by AF64A, a cholinotoxin.

## 2. Materials and Methods

### 2.1. Plant Materials and Preparation

Fresh leaves of *M. oleifera* were collected during November–December 2010 from Khon Kaen province, Thailand. After the authentication, the herbarium specimen was kept at Integrative Complementary Alternative Medicine, Khon Kaen University (voucher specimen 2010001). The fresh leaves were immediately cleaned, cut into small pieces, and dried in oven at 40°C. The dried plant material was ground into powder and extracted with 50% hydroalcohol (50% water : 50% alcohol) in a flat bottom flask at room temperature and allowed to stand for several days with occasional shaking. When the solvent becomes concentrated, the content is then filtered through Whatman number 1 filter paper and then concentrated with rotator evaporator at 45°C, dried, and kept at 4°C till used for further study. The yielded extract was 17.49% and contained total phenolic compounds and flavonoids at concentrations of 62 ± 0.08 mg of GAE·g^−1^ extract (milligram of Gallic acid equivalents) and 29.9 ± 0.02 mg QE/g extract (milligrams of Quercetin), respectively.

### 2.2. Animals

Male Wistar rats, weighing 180–220 g, were used as experimental animals. They were obtained from National Animal Center, Salaya. They were housed 6 per cage and maintained in 12 : 12 light : dark cycle and given access to food and water ad libitum. The experiments were performed to minimize animal suffering and the experimental protocols were approved by the Institutional Animal Care and Use Committee, Khon Kaen University, Thailand (AEKKU 41/2554).

### 2.3. Surgical Procedures

The animals were anesthetized by intraperitoneal injection of sodium pentobarbital at dose of 60 mg/kg BW. Then, AF64A (2 nmol/2 *μ*L) was infused bilaterally via intracerebroventricular (i.c.v.) route with a 30-gauge needle inserted through a burr hole drilled into the skull into both the right and left lateral ventricles. Stereotaxic coordinates were measured (from the bregma): posterior 0.8 mm, lateral ±1.5 mm, and ventral (from dura) 3.6 mm. The rate of infusion is 1.0 *μ*L/min. The needle were left in place for 5 min after infusion and then slowly withdrawn [20].

### 2.4. AF64A Administration

AF64A was prepared as described previously [20, 21]. Briefly, an aqueous solution of acetylethylcholine mustard HCl (Sigma, St. Louis, MO) was adjusted to pH 11.3 with NaOH. After stirring for 30 min at room temperature, the pH was lowered to 7.4 with the gradual addition of HCl and stirred for 60 min. The amount of AF64A was then adjusted to 2 nmol/2 *μ*L. The vehicle of AF64A was distilled water prepared in the same manner as the AF64A and recognized as artificial cerebrospinal fluid (ACSF) [20, 21].

### 2.5. Experimental Protocol

**Figure 1:**
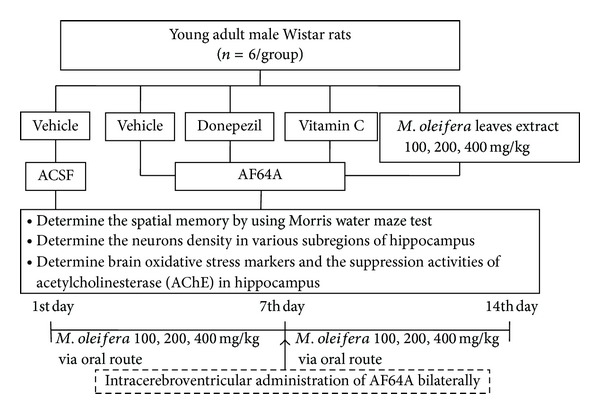
Schematic diagram shows the experimental protocol.

All rats were randomly assigned to 7 groups of 6 animals each as follows: (1) vehicle + ACSF: rats received vehicle via oral route and received artificial cerebrospinal fluid (ACSF) via intracerebroventricular (i.c.v.) route bilaterally; (2) Vehicle + AF64A: rats received distilled water (vehicle) via oral route and received AF64A, a cholinotoxin, via the intracerebroventricular route bilaterally; (3) Donepezil + AF64A: rats had been given Donepezil (1 mg/kg BW) via oral route 7 days prior to the administration of AF64A bilaterally via intracerebroventricular route and 7 days after AF64A administration via the intracerebroventricular route bilaterally; (4) Vitamin C + AF64A: rats received Vitamin C (250 mg/kg BW) at a period of 7 days before and 7 days after AF64A administration via the intracerebroventricular route bilaterally; (5)–(7) *M. oleifera* 100, 200, and 400 mg/kg: rats in these groups were given the extract at one of the following doses of 100, 200, and 400 mg/kg via oral route 7 days before AF64A administration bilaterally via intracerebroventricular route and the extract was continually administered for 7 days after AF64A administration. All rats were determined spatial memory using Morris water maze test at the end of experiment. Then, they were sacrificed and brains were isolated to determine the neurons density in various sub regions of hippocampus. In addition, brain oxidative stress markers and the suppression activities of acetylcholinesterase (AChE) in hippocampus were also evaluated as shown in Figure 1.

### 2.6. Determination of Spatial Memory

Spatial memory was evaluated via the Morris water maze. The water maze consists of a metal pool (170 cm in diameter × 58 cm tall) filled with tap water (25°C, 40 cm deep). The pool was divided into 4 quadrants (Northeast, Southeast, Southwest, and Northwest). The water surface was covered with nontoxic milk. The removable platform was placed below the water level at the center of one quadrant. For each animal, the location of the invisible platform was placed at the center of one quadrant and remained there throughout training. The times for animals to climb on the hidden platform were recorded as escape latency. In order to determine the capability of the animals to retrieve and retain information, the platform was removed 24 hr later and the rats were released into the quadrant diagonally opposite to that which contained the platform. Time spent in the region that previously contained the platform was recorded as retention time [22].

### 2.7. Histological Procedure

After the anesthesia with sodium pentobarbital (60 mg/kg BW), brains were subjected to transcardial perfusion with fixative solution containing 4% paraformaldehyde in 0.1 M phosphate buffer pH 7.3. After the perfusion, brains were removed and stored over a night in a fixative solution used for perfusion. Then, they were infiltrated with 30% sucrose solution at 4°C. The specimens were frozen rapidly and 30 *μ*M thick sections were cut on cryostat. The selected sections were rinsed in the phosphate buffer and picked up on slides coated with 0.01% of aqueous solution of a high molecular weight poly-L-lysine.

### 2.8. Morphological Analysis

Five coronal sections of each rat in each group were studied quantitatively. Neuronal counts in hippocampus were performed by eye using a 40x magnification with final field 255 *μ*m^2^ according to the following stereotaxic coordinates: AP −4.8 mm, lateral ±2.4–6 mm, and depth 3–8 mm. The observer was blind to the treatment at the time of analysis. Viable stained neurons were identified on the basis of a stained soma with at least two visible processes. Counts were made in five adjacent fields and the mean number extrapolated to give total number of neurons per 255 *μ*m^2^. All data are represented as number of neurons per 255 *μ*m^2^.

### 2.9. Determination of Malondialdehyde Level and Acetylcholinesterase Activity

Hippocampus was isolated and prepared as hippocampal homogenate and the determination of the malondialdehyde (MDA) level and acetylcholinesterase (AChE) activity in hippocampus were performed. Malondialdehyde was indirectly estimated by determining the accumulation of thiobarbituric acid reactive substances (TBARS) [23] while the activity of AChE was determined using Ellman method [24].

### 2.10. Determination of Scavenging Enzymes Activities

In order to determine the activities of antioxidant enzymes including superoxide dismutase (SOD), catalase (CAT), and glutathione peroxidase (GSH-Px), the brains tissues were weighed and homogenized with a buffer consisting of 10 mM sucrose, 10 mM Tris-HCl, and 0.1 mM EDTA (pH 7.4). Then, the brain homogenates were centrifuged at 3000 g for 15 min at 4°C. The supernatant was used for bioassays. The activity of SOD was determined using a xanthine/xanthine oxidase system for the production of superoxide radical and subsequent measurement of cytochrome *c* as a scavenger of the radicals. Optical density was determined using a spectrometer (UV-1601, Shimadzu) at 550 nm [25]. One unit of enzyme activity was defined as the quantity of SOD required to inhibit the reduction rate of cytochrome *c* by 50%. SOD activity was presented as units per milligram of protein (U mg^−1^ protein). CAT activity in the supernatant was measured by recording the reduction rate of H_2_O_2_ absorbance at 240 nm [26]. The activity of CAT was expressed as *μ*mol H_2_O_2_/min/mg protein. GSH-Px was determined using *t*-butyl hydro peroxide as a substrate. The optical density was spectrophotometrically recorded at 340 nm [27]. One unit of the enzyme was defined as micromole (*μ*mol) of reduced nicotinamide adeninedinucleotide phosphate (NADPH) oxidized per minute. GSH-Px activity was expressed as U/mg protein.

### 2.11. Statistic Analysis

Data were expressed as means ± S.E.M. and analyzed statistically by one-way ANOVA, followed by post hoc (LSD) test. The results were considered statistically significant at *P*-value < .05.

## 3. Results

### 3.1. Effect of *M. oleifera* Leaves Extract on Spatial Memory

**Figure 2:**
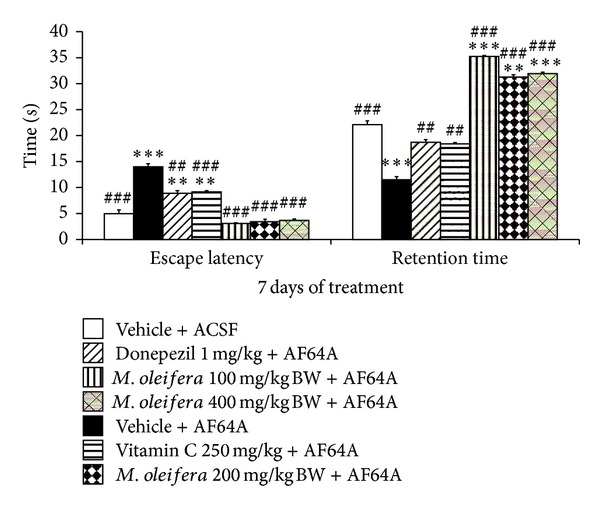
Effect of *M. oleifera* leaves extract on escape latency and retention time of memory deficit rats induced by AF64A, a cholinotoxin, in Morris water maze test. Each column and bar represent mean ± S.E.M. (*n* = 6/group). ^##^ *P* value< .01, ^###^ *P* value< .001 compared to vehicle + AF64A. **P value < .01, ***P value < .001 compared to vehicle + ACSF.

Based on the crucial role of cholinergic function and hippocampus on learning and memory mentioned earlier, we have induced memory impairment as that observed in dementia by using the bilateral administration of AF64A, a cholinotoxin, into lateral ventricle via intracerebroventricular route in order to induce cholinergic damage in the area around lateral ventricle especially hippocampus which in turn induces spatial memory impairment. In this study, we evaluated the spatial memory by using escape latency and retention time in Morris water maze as indices. The results were shown in Figure 2. It was found that AF64A administration significantly increased the escape latency but decreased retention time (*P*-value < .001 compared to vehicle + ACSF group). Both Donepezil and Vitamin C treatments significantly mitigated the enhanced escape latency (*P*-value < .01 and .001, respectively, compared to vehicle + AF64A group) and the decreased retention time (*P* < .01 all compared to vehicle + AF64A group) induced by AF64A. All doses of *M. oleifera* leaves extract also significantly mitigated the enhanced escape latency (*P*-value < .001 compared to vehicle + AF64A group) and the decreased retention time induced by AF64A (*P*-value < .001 compared to vehicle + AF64A group).

### 3.2. Effect of *M. oleifera* Leaves Extract on Hippocampal Neurodegeneration

**Figure 3:**
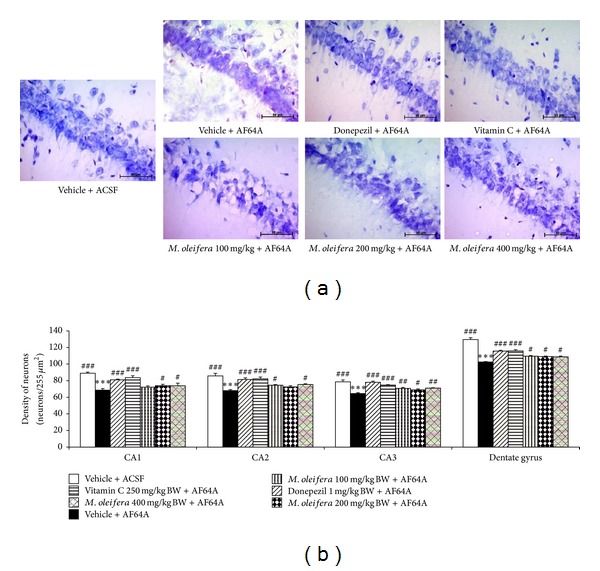
Effect of *M. oleifera* leaves extract on neurons density in various subregions of hippocampus of memory deficits rats induced by AF64A. (a) Image of neurons in CA1 of hippocampus stained with cresyl violet. (b) Neurons density in various sub-regions of hippocampus after various treatments including vehicle, Donepezil, Vitamin C, and various *M. oleifera* leaves extract at doses of 100, 200, and 400 mg·kg^−1^ BW. The column and bar represent mean ± S.E.M. (*n* = 6/group) ***P < .001 compared to vehicle + ACSF group; ^#^, ^##^, ^###^ *P* < .05, .01, and .001, respectively, compared to vehicle + AF64A group.

Since memory impairment is associated with the neurodegeneration in hippocampus [28, 29], we also determined the effect of *M. oleifera* leaves extract on neurons density in various sub-regions of hippocampus. The results were shown in Figure 3. It was found that AF64A significantly decreased neurons density in CA1, CA2, CA3, and dentate gyrus (*P*-value < .001 all; compared to vehicle + ACSF group). Both Donepezil and Vitamin C could mitigate the decreased neurons density in all areas mentioned earlier (*P*-value < .001 all compared to vehicle + AF64A group). The crude extract of *M. oleifera* at high concentration (400 mg kg^−1^ resp.) significantly attenuated the reduction of neurons density in CA1, CA2, CA3 and dentate gyrus (*P*-value < .05, .05, .01 and .05 respectively; compared to vehicle + AF64A group) while the medium concentration (200 mg kg^−1^) produced a significant attenuation effect on the decreased neurons density in CA1, CA3 and dentate gyrus (*P*-value < .05 all compared to vehicle + AF64A group) and low dose concentration produced an attenuation effect on the decreased neurons density in CA2, CA3, and dentate gyrus (*P*-value < .05, .01, and .05, respectively, compared to vehicle + AF64A group).

### 3.3. Effect of *M. oleifera* Leaves Extraction Oxidative Stress Markers and AChE Enzyme Activity

**Figure 4:**
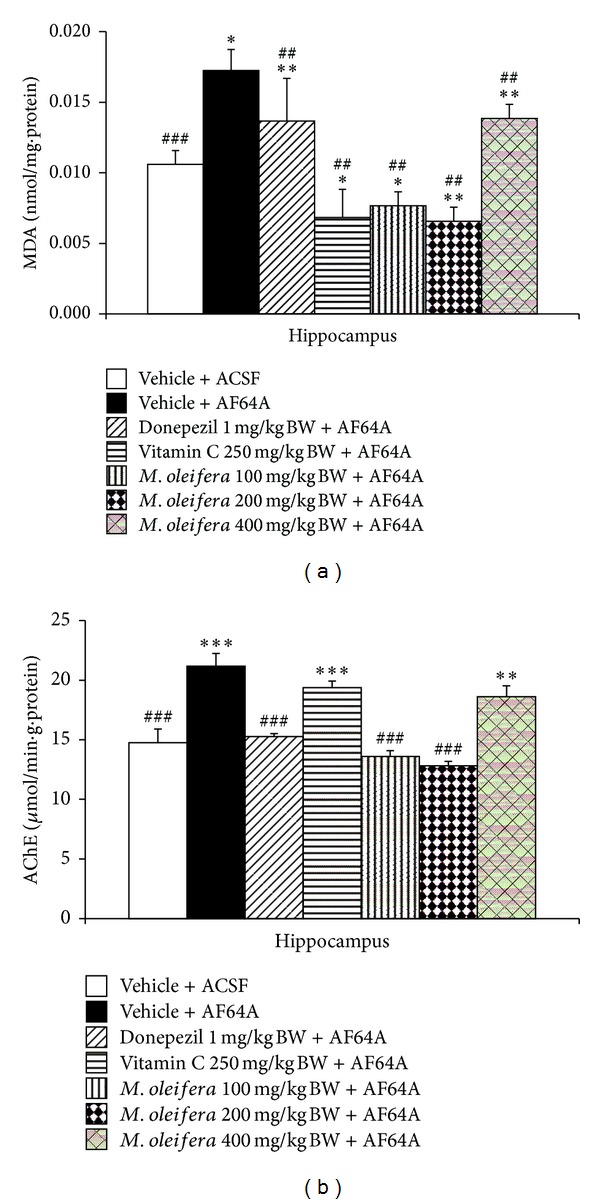
Effect of* M. oleifera* leaves extract on the level of malondialdehyde (MDA), a lipid peroxidation product, and the activity of acetylcholinesterase AChE enzyme in hippocampus. (a) Effect of *M. oleifera* leaves extract on malondialdehyde (MDA) level. (b) Effect of *M. oleifera* leaves extract on the activity of AChE. The column and bar represent mean ± S.E.M. (*n* = 6/group). *, **, ***P < .05, .01, and .001, respectively, compared to ACSF group. ^##^, ^###^ *P* < .01 and .001, respectively, compared to vehicle + AF64A group.

**Figure 5:**
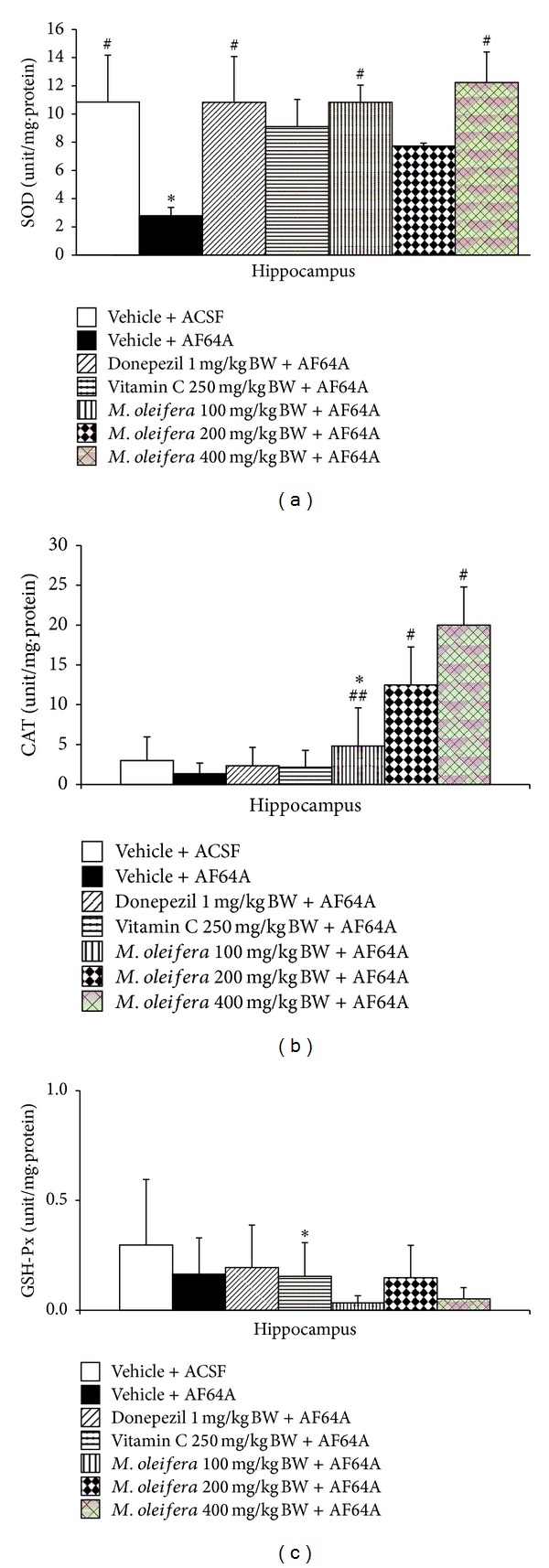
Effect of *M. oleifera* leaves extract on the activities of scavenger enzymes in hippocampus. (a) Effect of *M. oleifera* leaves extract on superoxide dismutase (SOD) activity. (b) Effect of *M. oleifera* leaves extract on catalase (CAT) activity. (c) Effect of *M. oleifera* leaves extract on glutathione peroxidase (GSH-Px) activity. The column and bar represent mean ± S.E.M. (*n* = 6/group). *, **, ***P < .05, .01, and .001, respectively, compared to ACSF group; ^#^, ^##^ *P* < .05 and .01, respectively, compared to vehicle + AF64A group.

Based on the crucial role of oxidative stress and cholinergic system function on memory impairment previously mentioned, this part of study was focused on the effect of *M. oleifera* on oxidative stress markers including MDA level and the activities of scavenger enzymes including SOD, CAT, and GSH-Px and on the activity of AChE, an indirect indicator reflecting the available acetylcholine, in hippocampus. The results were shown in Figures 4 and 5. It was demonstrated that AF64A injection significantly increased MDA level as shown in Figure 4 (*P* < .05 compared to ACSF + vehicle group) but decreased the activities of SOD (*P* < .05 compared to vehicle + ACSF) and CAT (*P* < .001 compared to vehicle + ACSF group) as shown in Figure 5. Interestingly, the elevation of MDA level in hippocampus was mitigated by Donepezil, Vitamin C, and all doses of *M. oleifera* leaves extract (*P* < .01 all compared to vehicle + ACSF group).

We also determined the effect of *M. oleifera* extract on the activities of main scavenger enzymes including superoxide dismutase (SOD), catalase (CAT), and glutathione peroxidase (GSH-Px) in hippocampus. Figure 5 showed that AF64A significantly decreased SOD and CAT activities whereas no change of GSH-Px activity was observed (*P*-value < .05 and .001, respectively, compared to vehicle + ACSF group). Vitamin C treatment could attenuate the reduction of SOD and CAT activities induced by AF64A (*P*-value < .05 all compared to vehicle + AF64A group). High dose of *M. oleifera* significantly attenuated the decreased activities of SOD and CAT induced by AF64A (*P*-value < .05 all compared to vehicle + AF64A group) whereas the low dose of extract could only produce a significant modulation effect to attenuate the decreased of SOD activity in the mentioned area. No other significant changes were observed as shown in Figure 5.

The effect of *M. oleifera* leaves extract on the activity of AChE in hippocampus was also investigated. The results were shown in Figure 4. It was found that AF64A enhanced AChE but it failed to show the significant effect. *M. oleifera* leaves extract at doses of 100 and 200 mg·kg^−1^ BW significantly decreased AChE activity in hippocampus (*P* < .001 and .01, resp. compared to vehicle + AF64A group).

## 4. Discussion

The current study has investigated the effect of *M. oleifera* leaves extract on spatial memory and on the neurodegeneration, oxidative stress markers, and the alteration of AChE activity in hippocampus. The results clearly demonstrated that *M. oleifera* leaves extract significantly improved spatial memory and decreased neurodegeneration in CA1, CA2, CA3, and dentate gyrus of hippocampus together with the decreased MDA level but increased SOD, CAT, and AChE activities.

Recent studies have demonstrated that dorsal hippocampus provides animals with a spatial map of their environment [30]. It makes use of reference and working memory and has an important role in information processing which involves spatial locations [31]. Lesion in this region results in problems concerning goal-directed navigation and impairs the ability to remember precise locations [32]. Various subregions of hippocampus play different roles in spatial memory. It has been demonstrated that lesion to the ventral hippocampus produces no effect in spatial memory and the dorsal hippocampus plays essential role in retrieval, processing short-term memory and transferring memory from the short term to longer delay periods [33–35]. The memory encoding process of spatial memory is associated with the major hippocampal subregions, CA1, CA3, and dentate gyrus (DG), especially in dorsal hippocampus [36], whereas the recall process is associated with CA3 [37, 38]. Unfortunately, the role of CA2 is still unclearly understood.

The data obtained from this study showed that *M. oleifera* treated groups showed the decreased escape latency but increased retention time. The changes were higher than those observed in Vitamin C and Donepezil treated groups. The possible explanation might be related to the multitarget sites of action of *M. oleifera*. Numerous factors are contributing to the important roles in memory retention. Besides cholinergic function and the density of neuronal cells which contribute to the crucial role in memory retention circuit in cerebral cortex, the increased cerebral blood flow and the increased dopaminergic function also contribute to the role in memory retention [39–41]. It was found that *M. oleifera* did not only suppress acetylcholinesterase (AChE) activity but also increased neurons density. In addition, *M. oleifera* also exhibits vasodilation effect [42, 43] and modulates the function of monoamines transmitters such as dopamine [43, 44]. Since *M. oleifera* exerts its effect on multi-target sites which contribute to the crucial role in memory retention, it could exert its influence more than Donepezil or Vitamin C treatments.

Recently, it has been reported that the neurodegeneration of hippocampal neurons is under the influence of oxidative stress [45, 46], calcium homeostasis disturbance [47], apoptosis [48], and the decreased vascular supply [49]. Since the interaction between various constituents in *M. oleifera* leaves extract could modify the bioavailability and signal transduction pathway of active substances in the extract, no dose dependent response manners of neuronal density changes, oxidative stress markers, and AChE activity were observed in hippocampus.

**Figure 6:**
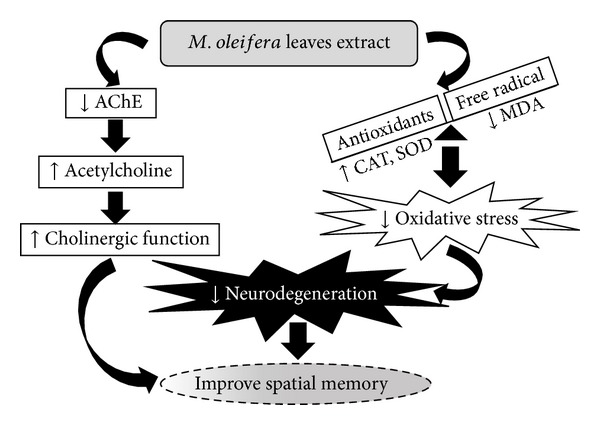
Schematic diagram shows the possible underlying mechanism of memory enhancing effect of *M. oleifera* leaves extract.

This study demonstrated that the memory enhancing effect of *M. oleifera* leaves extract might occur partly via the decreased oxidative stress and the enhanced cholinergic function. These effects have been shown in Figure 6. However, other mechanisms concerning the vasodilation effect [42] which in turn increased regional blood flow and the suppression of monoamine oxidase (MAO) which gave rise to the enhanced dopaminergic function [43] induced by *M. oleifera* leaves extract might also play the pivotal role in the cognitive enhancing effect of *M. oleifera* leaves extract. Based on the previous finding that the neuronal dysfunctions and neurodegeneration could be improved by flavonoids [50], we did suggest that the neuroprotective and cognitive enhancing effects of *M. oleifera* leaves extract might occur partly via the flavonoids in the extract. However, this still requires further investigation.

## 5. Conclusion

The current results suggest that *M. oleifera* leaves extract possesses the neuroprotective and memory enhancing effects. The possible underlying mechanisms appear to depend on application doses. Low and medium doses seem to provide beneficial effects just mentioned via the decreased oxidative stress and the suppression of AChE activity whereas high dose of extract appears to induce the beneficial effects primarily via the decreased oxidative stress. Since the effective doses are very much less than LD50, *M. oleifera* leaves extract may be served as the potential medicinal food against dementia. However, further explorations concerning active ingredient(s) and the detail of underlying mechanism of the extract are still required.
